# Dog alerting and/or responding to epileptic seizures: A scoping review

**DOI:** 10.1371/journal.pone.0208280

**Published:** 2018-12-04

**Authors:** Amélie Catala, Hugo Cousillas, Martine Hausberger, Marine Grandgeorge

**Affiliations:** 1 Université de Rennes, Normandie Univ, CNRS, EthoS (Éthologie animale et humaine)—UMR 6552, Paimpont, France; 2 Association Handi’Chiens, Paris, France; 3 Université de Rennes, Normandie Univ, CNRS, EthoS (Éthologie animale et humaine)—UMR 6552, Rennes, France; 4 CNRS, Université de Rennes, Normandie Univ, EthoS (Éthologie animale et humaine)—UMR 6552, Paimpont, France; Memorial University of Newfoundland, CANADA

## Abstract

Recently, there has been a rising interest in service dogs for people with epilepsy. Dogs have been reported as being sensitive to epileptic episodes in their owners, alerting before and/or responding during or after a seizure, with or without specific training. The purpose of this review is to present a comprehensive overview of the scientific research on seizure-alert/response dogs for people with epilepsy. We aimed to identify the existing scientific literature on the topic, describe the characteristics of seizure-alert/response dogs, and evaluate the state of the evidence base and outcomes. Out of 28 studies published in peer-reviewed journals dealing with this topic, only 5 (one prospective study and four self-reported questionnaires) qualified for inclusion according to PRISMA guidelines. Reported times of alert before seizure varied widely among dogs (with a range from 10 seconds to 5 hours) but seemed to be reliable (accuracy from ≥70% to 85% according to owner reports). Alerting behaviors were generally described as attention-getting. The alert applied to many seizure types. Dogs mentioned as being seizure-alert dogs varied in size and breed. Training methods differed between service animal programs, partially relying on hypothesized cues used by dogs (e.g., variations in behavior, scent, heart rate). Most studies indicated an increase in quality of life and a reduction in the seizure frequency when living with a dog demonstrating seizure-related behavior. However, the level of methodological rigor was generally poor. In conclusion, scientific data are still too scarce and preliminary to reach any definitive conclusion regarding the success of dogs in alerting for an impending seizure, the cues on which this ability may be based, the best type of dog, and associated training. While these preliminary data suggest that this is a promising topic, further research is needed.

## Introduction

The relationship between human and domestic animals has a long history. As a side effect of domestication [1] and/or shared life [2], it is generally assumed that domestic animals have developed abilities to perceive and interpret human signals. Thus, for example, miniature pigs are able to use visual cues to discriminate between people [3], horses have demonstrated the ability to perceive human attentional or emotional states [4,5] and goats follow gaze direction and use human communicative cues to find hidden food [6]. In particular, dogs were the first species to be domesticated [7] and have been selectively bred for traits relevant to interactions with humans, such as tameness and sensitivity to human cues, as well as specific skills related to herding, hunting or companionship [8]. Dogs are a species that naturally live with human families [9], which may facilitate the development of different social-cognitive skills [10,11]. They are able to understand human social and communicative behavior (e.g., to find hidden treats) [12] and are sensitive to human attentional states [13–16]. In addition, dogs are responsive to humans’ emotional expressions and to emotional cues conveyed by the human face [17–21]. Dogs also seem to be sensitive to human olfactory signals, behaving differently according the person’s emotional state [22,23].

According to U.S. legislation, a “service dog” is a dog individually trained to perform tasks for the benefit of an individual with a disability [24]. For example, guide dogs assist blind people in avoiding obstacles, hearing or signal dogs alert their hearing impaired owners to the sounds of a telephone or fire alarm, while mobility assistance dogs help people with mobility impairments to bring objects that are out of reach, pull wheelchairs or open doors, among others tasks.

More types of service dogs have been established recently, such as service dogs for children with autism spectrum disorders, which are supposed to help calm them, and to improve their skills (as prosociality) and safety [25,26]. We can also mention diabetes alert dogs that signal hypoglycemia episodes [27,28] and psychiatric service dogs, which support people with psychiatric disorders such as depression, panic and post-traumatic stress [29,30].

More recently, there has also been an interest in service dogs that could help people with epilepsy: seizure dogs. Epilepsy is a neurologic disorder characterized by recurrent seizures [31]. Seizures are divided into three main categories: generalized, focal and unknown onset seizures. Generalized onset seizures (such as tonic-clonic, absence or atonic) affect both cerebral hemispheres from the onset of the seizure and induce a loss of consciousness, while focal seizures affect an area of one hemisphere. Focal onset seizures are subdivided into focal onset aware seizures (simple partial seizures), where consciousness is retained, and focal onset impaired awareness (complex partial seizures), where consciousness is impaired [32]. For 20 to 30% of patients, seizures are either intractable or uncontrolled or they suffer from significant adverse side effects to medication [33]. Various comorbid psychiatric disorders are commonly associated with epilepsy such as anxiety and depression [34–37]. Depression is possibly related to biological factors (effects of seizures on mood regulation, effect of the anti-epileptic drugs, family predispositions for mood disorder, etc.) as well as to psychosocial factors (fear of experiencing seizure, stigmatization, etc.) [38,39]. Persons with epilepsy were more likely to experience psychological distress such as feelings of sadness, nervousness, hopelessness, worthlessness and undertake insufficient leisure-time physical activity [40].

Interestingly, in the early 1990s, reports of pet dogs that were spontaneously sensitive to the epileptic episodes of their owners were published in a lay publication for dog fanciers [41]. A first pilot study followed, suggesting that dogs could be trained to recognize seizures [42]. Surveys were designed to study seizure alert and response dogs, mostly in pets but also through specifically trained service dogs [43–45]. Some authors [46–48] tried to assess the seizure-alerting abilities of dogs through video electroencephalographic (video-EEG) recording with case studies, but results were mostly inconclusive due to the presence of psychogenic non-epileptic seizures (PNES) instead of epileptic seizures. For two persons with epileptic seizures, though no data were presented, it has been reported that dogs (one possibly trained [48] and one pet [47]) were able to alert their owners. Three recent reviews exist on this specific subject [49–51].

Across the world, some associations train dogs to assist such people. Two types of service dog are considered for people with epilepsy: seizure-alert dogs (SAD), which seem to anticipate seizures and warn their owner accordingly, allowing him/her to seek out a safe place or to take any action that could get him/her to safety; and seizure response dogs (SRD) which demonstrate specific behaviors during or immediately after a seizure. A parallel can be made between inanimate seizure detection devices that aim to detect ongoing seizures and SRD as well as between prediction systems, which must be able to identify pre-ictal modifications and SAD [52]. To date, seizure detection devices seems to reach reliability as technology advances, although prediction devices are still at early stages [53,54]. In all cases, systems will probably need to be individualized to be optimal, as a selection or combination of specific devices will suit some type of seizure more than others [54,55].

Dogs can have spontaneously acquired SAD skills or have been specifically trained for this purpose (for example, seeking help in case of an emergency, that dogs would possibly not do without training [56]). However, as highlighted in previous reviews [51,57], there is at this stage a lack of rigorous clinical trials to confirm these abilities and inform more about the mechanisms that are potentially involved.

These assertions raise the question of the impact of such a dog on the quality of life of a person with epilepsy. It is in general widely accepted that dogs may have beneficial effects on various aspects of daily life [58–61]. Positive effects can be found at a psychosocial level, pet ownership being positively associated with some forms of social contacts and interactions and with perceptions of neighborhood friendliness [62,63]. Pet ownership can also improve mental and physiological health status [64]. Several studies suggest that it can positively impact on stress [65,66], in particular for highly stressed or socially isolated individuals. Interacting with a pet has been reported to reduce anxiety [67,68] and depression [69–71] and to enhance the quality of life [71–73].

Focusing more on assistance animals, it has been suggested that in addition to helping with the task they have been trained for, they can improve the psychosocial health of their owners by reducing anxiety level [74,75], depression and feelings of loneliness [76–78] as well as increasing their social support [75,77,79], self-esteem [76,78] and general quality of life [80]. It has also been reported that persons living with an assistance dog experienced enhanced perceptions of health, independence and feelings of safety [74,75,77–79]. Considering the various comorbid associated disorders (e.g., anxiety, depression), one could argue for the presence of a pet in a person with epilepsy’s home.

The purpose of this review is to present a comprehensive overview of the validated scientific research on dogs that help people with epilepsy, in order to go beyond the anecdotal level. A scoping review appeared to be more designed to answer broad questions, contrary to a systematic review, as we aimed to identify, summarize and evaluate all existing empirical studies on such dogs in order to document practices and their reported findings, as well as to provide directions for further, more rigorous research. The specific goals are to (a) describe the characteristics of seizure-dogs, (b) evaluate the state of the evidence base, and (c) summarize the reported outcomes of the use of these dogs.

## Methods

We followed the Joanna Briggs Institute guidelines on conducting systematic scoping reviews [81–83]. This methodology summarizes the evidence available on a topic in order to convey the breadth and depth of that topic.

### Protocol

Although traditionally applied to systematic reviews, the PRISMA Statement was used to perform this scoping review [84] as followed in a recent review on a similar research topic [85]. A study protocol that specified the search strategy and inclusion and exclusion criteria was defined a priori.

### Information sources and search strategy

Studies were identified by searching the following electronic databases from their inception date through December 2017: ERIC (1966 –Present), Medline (1950 –Present), PsycARTICLES (1987 –Present), PsycINFO (1806 –Present), and Scopus (1960 –Present). To increase coverage, an additional database was included: HABRI Central (Human-Animal Bond Research Initiative), a specialized human-animal interaction research database. Search terms for all databases included at least one identifier for seizure and at least one identifier for seizure-dogs in the article title, abstract, and/or keywords. Identifiers for seizure included epilepsy or seizure. Identifiers for seizure-dogs included the terms alert dog, detection dog, service dog, assistance dog, response dog, intervention dog, prediction dog and dog. For all articles meeting the inclusion criteria, reference lists were screened for possible additions.

### Eligibility criteria

The following inclusion criteria were used to select relevant articles for review: (a) publication in English in a peer-reviewed journal, (b) concerned with outcomes from the use of seizure-dogs, which was defined as living with a dog said to alert and/or respond to seizures–whether spontaneously or after specific training, and (c) reporting of quantitative results for participants who have experienced seizures.

### Charting the data

Information was extracted from each included study to achieve the three aims of this scoping review. To achieve the first aim–describe key characteristics of a seizure-dog—data items included alerting behaviors, alert time, dog characteristics, epilepsy characteristics and, when appropriate, training method. To achieve the second aim–evaluate study methodology and risk of bias—data items included sample size, participant characteristics (including age, gender and epilepsy diagnosis), study design, comparison condition and assessment measures (including standardized instruments, and raters/informants). To achieve the third aim–summarize study outcomes—data items included the results of each study, which were subsequently organized by the most commonly reported outcomes. Additional data items were extracted for study identification and exploratory purposes, including first author, year, country of corresponding author and journal name. Data were extracted by one team member and verified by a second reviewer.

## Results

### Study selection

**Fig 1:**
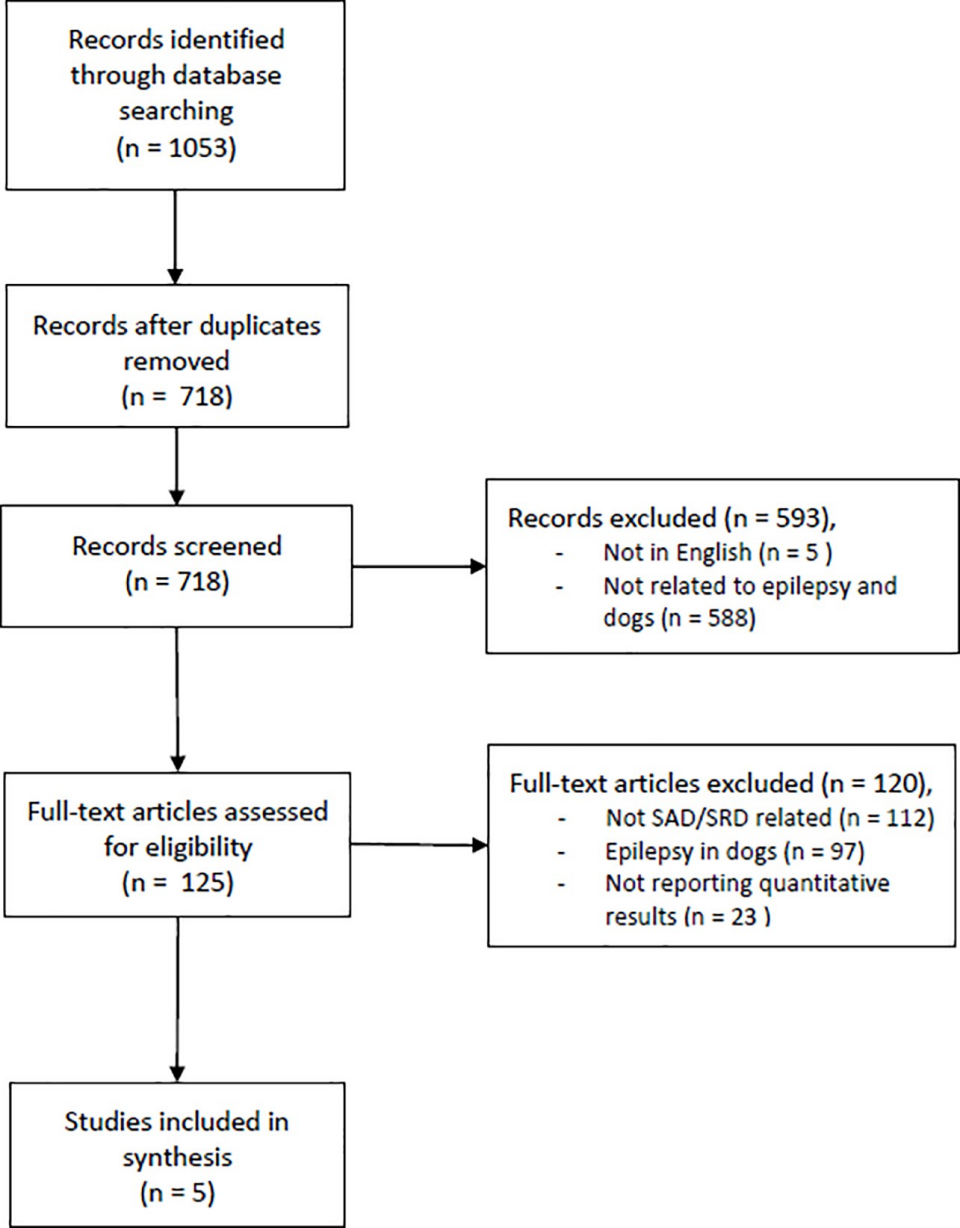
Flow chart of study selection process. SAD, seizure-alert dog; SRD, seizure-response dog.

The initial literature search resulted in 1053 citations. A flow diagram of the study selection process is presented in Fig 1. The final sample included 5 studies (Table 1) among which was 1 prospective study and 4 self-reported questionnaires or interviews. Twenty three studies were excluded due to lack of quantitative results, but are listed (Table A in S1 File) and some are discussed in S1 File.

**Table 1:** Summary of participants, study design, and outcomes of the five studies included in the scoping review: SAD, seizure-alert dog; SRD, seizure-response dog; ↑ greater, ↓ less,—not reported.

First author [reference]	Year	N (humans)	Human Age (years)	Human Gender (% male)	N (type of dogs)	Dogs training status	Comparison condition(s)	Outcomes
**Strong [86]**	2002	10	21–39	40	10 SAD	Trained	None	↓ Seizure frequency with SAD
**Dalziel [43]**	2003	9	18–60	56	6 SRD and 3 SRD + SAD	Untrained	Patients without alerting/responding dogs	Dogs may spontaneously alert and/or respond to seizures trend on seizure type success depend on the person's awareness and response to the dog's alerting behavior
		13 dog training centers	-	-	15 SRD + SAD	Trained	-	-
**Kirton [44]**	2004	10 with SRD and 10 with SRD+SAD	6.8–17.5	40 SRD 30 SAD	13 SRD and 9 SRD+SAD	Untrained	Patients without dog, patients with dog without behavior, patients with SRD	~15% SAD in the population surveyed Anticipation sensitive and specific ↑ Quality of life with a dog sensitive to seizures
**Kirton [45]**	2008	22	12–66	27	22	Trained	Retrospective survey (before owning the dog)	↑ Quality of life SRD developed SAD skills in 59% of cases
**Kersting [87]**	2009	9	-	-	9	Trained	Answers reflecting "do not know/cannot judge" impression of the owners	Effective predictions ↓ Number of seizure ↑ well-being,

All selected studies were published in peer-reviewed journals. Publication dates ranged from 2002 to 2009. Countries of corresponding authors included the USA (1 study), UK (1 study), Germany (1 study) and Canada (2 studies). The articles were published in medical journals (4 studies, of which 3 in epilepsy-related journals and 1 in a neurology journal) in addition to a veterinary behavior journal (1 study).

The final sample of articles included studies with a range of designs, participant age groups, intervention types, and outcome measures. Due to heterogeneity across studies, the results of this review focus on descriptive and qualitative synthesis rather than meta-analysis.

### Seizure-dogs characteristics—alerting behaviors

Most alerting behaviors, whether spontaneously exhibited by the dog or learned through specific training, consisted of behaviors described as attention-getting behaviors. Service and pet dogs are described as displaying intense staring [43,45], whining [43,45] or anxious barking [45]. They can also show “close attachment” (not defined) [44,45], lick the face [45], pace in front of or around the owner [43], sniff, jump on or prevent the owner from leaving the house prior to seizures [45], and prevent the owner from standing up [44].

Data on pet dogs were collected through a survey by Kirton et al. (2004) [44]: the median reported alerting time was 2.5 (25th-75th percentiles: 0.9–15.0) minutes with a range from 10 seconds to 5 hours. The accuracy of alert was high (median sensitivity of 80% (25th-75th percentiles: 66–92) and there was no false alert (thus, specificity of 100%). Also in relation to pet dogs, another study reported an alerting time of approximately 3 minutes in advance of a seizure [43]. It has been stated that specifically trained dogs (service dogs) demonstrated alerting behaviors about 30 minutes on average before a seizure onset for tonic-clonic seizures but less (15 minutes) for complex partial seizures [86]. Finally, in a retrospective study of a training program for SRDs, dogs that developed an ability to alert for seizures showed alerting behaviors on average 31 minutes prior to ictal event (30 seconds to 3 hours) and 11 out of 13 (85%) subjects were reported with no missed events [45]. According to this study, whether service or pet, each dog appears to show specific and reliable alerting behaviors. Finally, timings of 30 seconds to 45 minutes prior to seizure are reported in Dalziel et al. (2003) [43], with an accuracy of ≥70% of the time for service dogs.

In general, dogs (service dogs as well as pet dogs) have been reported able to alert for complex partial and tonic-clonic seizures [43–45,86]. In particular, pet dogs seemed also able to alert for atonic and absence seizures [44]. Dalziel et al. (2003) [43] also suggest that dogs will be more likely to alert for complex partial seizures associated with migraines and/or auras/symptoms (patient-reported), such as indescribable weird feeling in their head, nausea, lip smacking or mouth movements, and changes in breathing.

Through the 5 articles included, some profiles can be found for the dog who responded and/or alerted to ictal events. In studies on untrained dogs (pet dogs) that presented alerting behaviors, 8 different pure breeds were reported: Golden Retriever, Standard Poodle, German Shepherd, Akita, Rough Collie, Rottweiler, Cairn Terrier and Great Pyrenees [44]. One additional dog was mixed-breed (Sheltie-Spitz). In addition, they noted that most alerting dogs were large-size dogs (7 out of 9, the remaining two being small dogs). These dogs were also mostly females (7 out of 9, i.e., 78%) but this may not be surprising, as females were the most represented in all groups (65% in non-responding to seizure dogs, 64% in seizure response dogs). In studies on specifically service dogs, the dogs that have been selected by trainers and presented alerting-behaviors were from five breeds: Labrador Retriever, Poodle, Border Terrier, Cocker Spaniel and Toy Poodle [45,87]. The most common breed group was Retrievers (n = 22), maybe because these breeds are already often used for other types of assistance. Consequently, Kirton et al. (2008) [45] also reported that the dogs selected by the trainers were generally large (mean weight: 28 kg). Among the studies reporting this information, the sex ratio seems to be approximately equal (55% of females in [45]). It should be noted that the number of dogs per breed presenting alerting behaviors is approximate in one of the studies [45]. While the number of dogs considered as seizure-dogs is given (22 dogs trained to respond to seizures and 13 spontaneously developing alerting behaviors following the training), breed distinctions are not specified. Nevertheless, even if this study is not considered in calculating the number of dogs per breed, there is still a majority of retrievers (n = 10).

According to trainers, dogs were mostly selected from rescue centers but some could come from specific breeding programs (no additional detail about these programs was presented) [43]. Dogs that entered a North American/Canadian SRD training program (Lions Club Foundation Dog Guides), came from a breeding program and were selected through a behavioral testing (without further detail) that included items such as disposition, control, distraction, responsiveness to human command, initiative, chase instinct, attention/sensitivity to the person, prolonged concentration or confidence [45]. The authors noted a “success rate” of 69% (of 32 dogs evaluated, 22 SRDs were trained through the study duration–duration not specified). Of those 22 SRDs, 13 have been reported as spontaneously developing a seizure alerting behavior. The onset was within 4 weeks for 46% (6/13) of them and evolved over months (not detailed) for others.

As mentioned above, it is difficult to have a precise idea of the real success rate in terms of predicting an episode, especially as the studies are scarce and often based on small samples. There is a high variability in the timing of the alert behaviors in relation to the time of the episode, which increases the difficulty in identifying the cues dogs used to predict it. Authors have suggested that dogs could be sensitive to human heart rate changes or to olfactory or visual changes (subtle behavioral modifications [86]) in the human subject, without excluding a multimodality hypothesis [43]. Of nine families with untrained SADs, two felt that that the mechanism of anticipation was related to smell and six that it was a unique sensory ability outside of the traditional five senses [44].

In general, all programs stated that skills were instilled through an operant conditioning method [45]. Among 13 Service Dog Training Centers interviewed [43], training methods varied but reward-based training techniques were applied if a dog began to alert in order to reinforce this behavior and training could take 6 months to 2 years to complete. Another study [86] highlighted the importance of socializing the selected dog with the human as first step and then mentioned the use of a reward-based operant conditioning paradigm to train the dog to recognize and alert for a seizure. The learning is maintained with intermittent positive reinforcement and assessed with video observations at the dog training center and at the patient’s home. A detailed procedure for Lions Club Foundation Dog Guides was reported [45]. Dogs came from a breeding program (said, without further detail, to have been “engineered toward favorable assistance work characteristics while minimizing recessive disease traits in accordance with established guidelines”) and, after being placed in foster homes until 12–14 months for socialization and basic skills training, received the behavioral testing mentioned previously. If assessed as suitable, the animals entered a 2–3 month period of intensive obedience training for assistance skills, and then were more specifically given general response training (including barking on command, fetching of assistive devices, and activation of emergency response systems). Once placed with their owners, the development of seizure-alerting capacities could appear spontaneously for some dyads. Similarly, most Service Dog Training Centers interviewed [43] (without specifications of a precise number) offered no guarantees that a dog would alert. In accordance with the patient’s needs, dogs were trained to perform tasks such as activating an alarm, supporting the patient until the end of the episode or wearing a backpack containing medication and emergency contact numbers. In addition, dogs were trained to alert a caretaker of an ongoing seizure (“helping the human companion to a safe place or position prior to or after a seizure”), although no further detail is reported on this training, with no information on the scientific or anecdotal basis of the process. An additional training was carried out if a dog began to alert to an impending seizure, in order to reinforce this behavior. According to Dalziel et al., the effectiveness of a SAD partially depended on the person’s ability to recognize and respond to the dog’s alerting behaviors [43]. It is also mentioned that one constant requirement through training facilities was future owner’s willingness and ability to bond with a dog.

### Methodological evaluation

To achieve the second aim, assessing methods and potential biases, key characteristics of the methods were extracted and summarized with respect to each study’s sample size and characteristics, study design (Table 1), and assessment type (Table 2).

**Table 2:** Assessment measures of the studies included in the scoping review: Standardized instruments and raters refer to survey; IPES, Impact of Pediatric Epilepsy Scale.

First author [reference]	Year	Study type	Standardized instruments	Raters/informants
**Strong [86]**	2002	Seizure diaries	-	Self
**Dalziel [43]**	2003	Survey	Based on [88–90]	Self
**Kirton [44]**	2004	Survey, telephone interview	IPES (impact of pediatric epilepsy scale)	Parents or self
**Kirton [45]**	2008	Survey/interview (phone, personal)	Based on [91]	Self or parents
**Kersting [87]**	2009	Survey	-	Self

Sample sizes ranged from 9 to 22 participants, with more than half of the studies (3/5) having a relatively small sample size of <11 participants. The percentage of males ranged from 27 to 56%. One study did not specify the participants’ gender. One study was conducted with children (n = 20), 2 with adults (n = 19), and one with both children and adults (n = 22). Age of participants ranged from 6.8 to 66 years. One study did not specify the age of participants. Two studies did not report the age range of their participants nor provide enough information to calculate it.

The participants included in all 5 studies had been diagnosed with epilepsy. Only one study has as an exclusion criteria epilepsy with complications, such as PNES (Psychogenic Non-Epileptic Seizures) [86]. One study did not provide any information on the type of seizure the participants had [87]. Among all considered studies, complex partial seizures were the most represented (51.3%) with tonic-clonic seizures as the second most prevalent (31%). Absence seizures and atonic respectively counted for about 5.1%. Myoclonic (3.8%). Atypical absence (2.56%) and simple partial seizures (1.28%) were the least represented seizure types.

None of the studies included a comparison condition, and only one looked at the treatment condition, using a pre-post design and a follow-up assessment [86].

Surveys and interviews were the most frequent means of assessment (n = 4), and the last article was based on seizure diaries. Responses to surveys were predominantly self-reports but could also include reports from parents or primary caretakers [44,45]. No study incorporated blinded observational nor physiological measures of participant outcomes.

### Outcomes of the presence of a seizure-dog

In one study [44], families with a child with epilepsy who had a dog scored themselves significantly higher in IPES (Impact of Pediatric Epilepsy Scale [92] Overall Quality of Life Score) than similar families without a dog. Still in comparison to families without a dog, this effect was even higher when dogs demonstrated seizure-related behavior. Nevertheless, within families that owned a dog, no significant difference in quality of life (QOL) score has been reported according to whether the dog had shown a seizure-related behavior during a seizure or not. Various studies indicate an increase in quality of life when living with a dog demonstrating seizure-related behavior [43–45,87]. In Kirton et al. 2008 [45], owners of specifically trained SRD reported improved overall QOL, clearly major in 82% of the cases and moderate in 18%. In a survey of 9 SAD owners [87], the presence of dogs seemed to improve their quality of life as they reported increases in perception of safety, social contact and going out compared to “do not know” or “they do not” answers.

In addition to alert and/or respond to seizures, it has been suggested that the presence of an assistant dog could affect seizure frequency and severity. According to Kirton et al. [45], 45% of patients reported an improvement. Strong et al. [86] conducted a prospective study on 10 patients in which tonic-clonic seizure frequency was monitored over a 48-week period, including a 12-week baseline after entry (subjects being their own control), a 12-week training period and 24-week follow-up. They noted that when comparing baseline seizure frequency to the last 12 weeks of follow-up, there was an average reduction of seizures by 43%. However, use of seizure diary self-report for this study and considerable inter-individual variability tend to moderate these results. Finally, in the Kersting et al. [87] survey on 9 patients, owners reported that their SAD had an impact on seizure frequency, as they stated their number of seizures decreased.

## Discussion

We conducted a scoping review to synthesize the empirical literature on dogs for people with epilepsy. The exhaustive search procedure resulted in 5 studies, all being peer-reviewed journal articles. This very weak number of studies on the subject that were the only ones to include quantitative data should highlight the urgency of more research in this field. In general, there has been a short period of interest in the topic (2002–2008), followed by large apathy in the literature, at least when considering studies that report quantitative data. Results suggest short-term, subjective benefits of seizure-dogs, including reduced seizure frequency, and improved quality of life. Intervention procedures and research designs varied greatly, evidencing the preliminary nature of research in this area. The field of research is international though not interdisciplinary, with a global range of corresponding author countries but with journal disciplines centered on clinical research. The broad range of outlets highlights the need for synthesizing the literature in one place and mapping key concepts, types of evidence, and gaps in research, which was the purpose of this scoping review. Each study was reviewed to achieve three key aims: (a) describe the characteristics of seizure-dogs, (b) evaluate the state of evidence base, and (c) summarize the reported outcomes of seizure-alert dogs.

### Characteristics of seizure-dogs

To achieve the first aim of the review, several elements of dogs were examined in each study, including alerting behaviors, alert time and reliability, epilepsy characteristics, dog characteristics, selection criteria, hypothetical cues and procedures.

All studies were consistent in their description of alerting behavior as attention-getting behaviors. Some terms would benefit from specification and we recommend that future studies define behaviors precisely to avoid risks of confusion.

The timing varied widely across studies, with some reports stating that it could depend on the type of seizure that the persons are experiencing [42,86], but it does not appear clearly through this review. Timing is very inconsistent across studies and not reported accurately by type of seizure, and therefore it would be difficult to make comparisons between studies. It seems that alerting behaviors appeared less in advance (2.5 and 3 minutes [43,44] prior to seizure vs. 15, 30 and 31 minutes [45,86]) in pet dogs than in trained dogs, but the variability range is definitely too wide to draw conclusions. In two additional studies, (see S1 File) that involved video-EEG monitoring and people with epilepsy [47,48], results differ largely, the first one reporting a 2second-before-seizure alert with 7 of 8 events missed, whereas the second one reported a 10- to 60-minute alert with no missed event. The only consistent point is that no study reported any false alerts. It is possible that at least part of the variability can be due to the complexity of characterizing the onset of a seizure in some cases. It would be of great interest to go further with this approach by including a larger number of SAD and people with epilepsy in a study with video-EEG control in order to assess the alerting abilities of dogs, their reliability, and characteristics.

Four studies reported details on epilepsy type that were alerted by dogs. All concluded that dogs (pet and service) can alert for complex partial and tonic-clonic seizures. Given the broad range of epilepsy types, it would be valuable for further research to investigate dogs’ alerting abilities according to these specificities. In addition to the type of epilepsy, factors that may influence the alert could for example include the presence or absence of auras, migraines, the cause of epilepsy (e.g., idiopathic, cryptogenic) and individual behavioral specificities. Given predominantly small sample sizes in existing studies, it is not possible to explore differences by category of epilepsy to determine the characteristics of seizures that can be alerted and thus characteristics of persons with epilepsy who benefit from SAD. Larger studies should be used in the future to distinguish the profiles of individuals who are most likely to be alerted and thus be most suitable for inclusion by Service Dog Training Centers, in addition to providing some directions for further research on mechanisms of the alert.

Untrained SAD (i.e., pet dog that spontaneously developed seizure alerting behaviors) included various breed types. Only two studies, both Canadian, described breeds of seizure- dogs. Among 8 different pure breeds reported, 3 breeds were of high prevalence in the Canadian dog population: Golden Retriever, Standard Poodle, German Shepherd [93]. Service dog breeds (n = 5) showed a similar distribution with 3 breeds highly frequent: Labrador Retriever and Poodle (Standard and Toy).

Details about dogs (e.g., breed, sex, age) in some studies were insufficient to enable correct characterization of pet dogs that spontaneously demonstrated seizure-alerting behaviors or a comparison between the characteristics of a spontaneously alerting dog and a dog that underwent training before beginning to alert. In general, all sizes and both sexes were represented. As for epilepsy types to which dogs responded, we recommend larger studies to assess reliability in dogs’ profiles.

Here again details are missing on traits or characteristics on which training programs based their choices for selecting future service dogs. Only one study [45] reported behavioral items considered for future service dog selection but did not describe them nor how they influenced the dog choice. It has been stated in other studies that priority was given to behavioral criteria and the apparent capacity of the dog to bond with humans rather than to gender, age or breed [43,49,94]. Further research should report these features and, when describing characteristics, carefully define each one.

Despite the possible priority given to dogs’ bonding abilities with owners when selecting dogs, a close bond between the dog and human may not be mandatory for seizure alerting, as one study mentioned that dogs have been reported to alert accurately to strangers (without further details) [43]. Consideration will need to be given as to whether this characteristic will be of use in the general role of a service dog or specifically in order to set up a reliable alerting behavior.

All studies were consistent in that they each stated that, at this time, there is no certainty about what cue a dog could use to predict a seizure. Studies showed that changes in heart rate can precede the onset of a clinically observable seizure by seconds or minutes [55,95,96] but the patterns of change might be individual-specific [95,97,98]. Olfactory cues could result from neurotransmitters and hormones released during an epileptic seizure. Nervous cell activity being impaired, body smell could be modified. This would be comparable to modifications described in connection with hypoglycemia in diabetes, for which dogs have been successfully trained [27]. Lastly, visual cues to an impending seizure could potentially be linked to people’s behavioral or postural modifications (e.g., changes in motor activity and or mood, experiencing auras). As mentioned earlier, dogs are quite able to detect such changes.

Interestingly, some patients report that they know when a seizure is going to occur and some studies have been carried out in order to assess these anecdotal reports. These patients often reported experiencing prodromes [99,100]. A prodrome is defined as a pre-ictal phenomenon, a subjective or objective clinical alteration that heralds the onset of an epileptic seizure but does not form part of it [101]. A prospective study [102], employing time-tracked data with personal digital assistants, questioned the predictive value of prodromes and the specificity of their occurrence in the pre-ictal period. Self-perceived prodromes could not significantly be used to predict a person’s own seizures. Nevertheless, it would seem possible that, using all their demonstrated human behavior reading skills [12,13,15], dogs could rely on occurrence of prodromes and their pre-ictal changes to alert to an impending seizure. In particular, it has been reported that these pre-ictal symptoms varied among patients and could occur between 5 minutes and 24 hours before the seizure [103]. Also, prodromes are cited in the context of complex partial seizures and generalized seizures [102–105]. Taken together, those aspects would fit with what has been reported in literature about seizure-alert dogs. It would even be possible that prodromes occur in a majority of patients but have not been perceived. Thus, it would be important to investigate the mechanisms involved in alerting behaviors and to find out which sensory modality or intermodality is involved. It would seem a logical second step of investigation after assessing the capacity of dogs to alert through video-EEG recording.

In general, dogs could come in various ways to a training program. Breeding programs are mentioned without precise details, but some dogs came from shelters or experienced a “career change” (e.g., previously trained as disability dogs but were no longer required in that role) [43,45,49]. Details regarding the training in seizure-alerting behaviors and the dogs’ preparation as well as practical details (e.g., is the dog rewarded if it alerts or when the seizure occurs) were globally missing, but in a review of *Support Dogs* (i.e., a U.K. charity) program [49], some aspects are developed. Brown and Goldstein [49] reported that the applicant enters the program beginning with an interview with training staff, who assess his or her commitment and ability to care for the dog and collect more information about the person. Then a two-day admission period allows an assessment of how he or she interacts with and reacts to dogs generally, and what types of dog the person may best bond with. If found satisfactory, the person is placed on a waiting list until a suitable dog is identified. There is then another check to see if the selected dog and applicant can bond with each other and if it is the case; there is a three-week admission period for the applicant to train the dog. Priorities are placed on bonding and on teaching the dog to focus intently on the person’s face (called “look at me” training). In parallel, when a seizure occurs, the dog is highly rewarded by the trainer. Depending on the frequency of seizures the dog may or may not acquire a seizure alert pattern after three weeks. Then, for two months, the dog and the person still follow the reward program at home and daily activities are recorded by video camera. A final week of admission allows a review of progress. The dog should be alerting to seizures by that time. Inclusion and exclusion criteria for applicants remain an open question [45,49] (e.g., minimum developmental age, minimum and maximum seizure frequency, type of seizure included, neurologist-confirmed epilepsy diagnoses, absence of PNES, other pets at home, etc.). It is noteworthy to consider the fact that, in most cases, Service Dogs Training Centers do not guarantee that a service dog for epilepsy will ever show alerting behaviors [43,45,48]. They train a dog to show seizure-responding behaviors, but the dog will have to spontaneously develop alerting abilities, and then this behavior will or will not be reinforced according to the training programs.

### Assessing seizure-dogs

To achieve the second aim of the review—to evaluate the state of the evidence base—we reviewed the methodology of the included studies.

There was some variability across the studies with respect to sample size and characteristics, study design, and assessments. Sample sizes ranged from 9 to 22 participants with seizure-sensitive dogs. Participants included children, adolescents, and adults. Not all studies reported descriptive statistics regarding participant age, as well as sex, type of epilepsy and dog characteristics (training status, breed, sex, age…), which should be more carefully reported in further research. The field would also benefit from larger sample sizes.

The type of epilepsy varied across studies, though two types were the most represented (tonic-clonic and complex partial seizures). It is noteworthy to consider that PNES (Psychogenic Non-Epileptic Seizures) might have been underrepresented in participants, as it was found in 3 of 4 case studies, implying SAD with video-EEG monitoring [46,48,106], and that dogs have already been shown alerting to a PNES [48]. According to the authors, this can raise problems as it may reinforce the idea in patients that they are going to have a seizure, which might in some cases even trigger a proper seizure. It does not mean that dogs are not able to alert to epileptic seizures in addition or instead of a PNES, but the alert can be misleading as it is not possible yet to have any understanding of the mechanisms implied and whether they are distinct between the epileptic seizure and the PNES. It would also be essential to determine whether the mechanisms are different depending on the type of epileptic seizures, and between epileptic and non-epileptic seizures. Given the small sample sizes in existing studies and the absence of data, it is not possible to document individual differences yet.

The trajectory of research to establish evidence-based and specialized SAD programs is in its very early stages. As do other reviews [50,51], we note that the current body of research consists predominantly of surveys or case studies. These types of studies do not allow a documentation of the efficacy, safety and potential benefits of living with a SAD, nor the best criteria in the choice of training (based on the mechanisms involved), nor the dogs that can be of interest, in relation to the cost of a service dog. In addition, surveys imply self-assessment of onset as well as frequency of seizures, assessments that have been proven difficult for patients [100,107]). This can put some doubt on the reliability of reports on dog’s seizure alerting abilities. Further studies should specifically assess and report characteristics for efficacy and safety, in addition to benefits and other parameters. More pre-post study designs can then follow, leading to the development of standardized procedures. However, we recognize that the steps to develop evidence-based, complementary, and integrative treatments are often not linear and there may be concurrent pursuits of multiple research goals.

One noteworthy area of consideration for further research is the selection of an appropriate control condition. According to Strong et al. (2000) [108], it would not be feasible to use a control group with pet dogs, as their health and welfare could be impaired as well as the security of the person with epilepsy. Nevertheless, in surveys such as the Kirton and al. (2004) [44] study, 48 families reported that they lived or had lived with a dog for at least 1 year while their child or children had seizures, without mention of any aggressive or fearful behavior from the dog. Though a self-reporting bias has to be taken into account, it would seem interesting to include people with epilepsy who live with pet dogs without aggressive or extremely anxious behavior as a control group. Indeed, in this review, none of the studies had control conditions. To investigate and provide evidence of the specific abilities of these dogs as well as their potential impact, a standard for control conditions is essential. More controlled studies would help to distinguish real impact from effects potentially due to novelty or expectancy biases.

Finally, the use of seizure diaries and surveys could lead to an important subjectivity bias. It is known that some seizures can be forgotten or occur in a state of unconsciousness [109], questioning the reliability of seizure diaries as seizure trackers [107,110]. In addition, the use of standardized surveys, allowing comparisons between studies, would be of interest.

### Outcomes of seizure-dogs

Although the reviewed studies are diverse and limited, a large majority reported positive outcomes of seizure-dogs for individuals with epilepsy. It is important to interpret these outcomes as preliminary, given the low level of methodological rigor in many of the studies. The most common findings were improved quality of life and a reduced number of seizures. This outcome is consistent with prior research mentioned in the introduction of this study, indicating that depression and epilepsy are two linked psychiatric disorders and highlighting the positive impact of pet ownership on the building blocks of depression (e.g., social isolation, stress, worthlessness…).

It remains unclear whether the effects described above with SRD and/or SAD are specifically linked to the assistance they provide, as the effects also correspond to findings on the human-dog relationship. In particular, as some studies suggest that the health-related quality of life (HRQOL) in people with epilepsy is more strongly associated with mood states than with either seizure frequency or severity [111,112], it could be a “simple” pet effect. Also, a potential “novelty effect” has to be considered, temporarily influencing frequency and severity of seizures. The impact of pet ownership on health seems to be most important for highly stressed or socially isolated individuals so it appears that an assistance dog could have a double effect—have the pet effect from one side, and by providing potentially a reliable warning of seizures, contribute to a decrease in anxiety and to an increase in confidence, independence and quality of life. Nevertheless, the specificity of this effect, as well as the pet effect in the context of epilepsy, remains yet unknown.

It would also be interesting to document the effect of a response dog compared to a pet dog and to a SAD, as well as the impact of the training on these outcomes or even the impact of the task that dogs–trained or not—are used to performing in the case of a seizure. Indeed, quite different behaviors are reported in the studies included in this review. Some of them are possibly not so helpful as a response to a seizure (e.g., face or hand licking, “protection behaviors” with barking at other people (e.g., see [113]), if not dangerous for caretakers. Thus, assessing the impact of these variations, and the relevance of trained versus untrained behaviors, can be of interest, as well evaluating whether the helpfulness and efficacy of dogs are maintained through time. As an example, some authors [45] initially postulated reliable behavior for all dogs, but then conceded that 13% of the dogs were not shown to be helpful during the night, and 22% had a poor response, or were unresponsive to seizures.

The duration of positive outcomes was only examined in one prospective study [86], which collected seizure frequency data for a further 24 weeks after the training period of SAD. The overall reduction in seizure frequency appeared to be maintained during this follow-up period. More research seems to be needed to assess with greater precision the impact of SADs on the life of their human partners with epilepsy.

Finally, it is notable that only few outcomes were reported related to animal welfare. One study stated that no adverse effect on canine health seemed to have been found [86], while another reported that behavioral problems were not uncommon in service dogs and that they may be prone to stress-induced illnesses [43] similar to those of working dogs [114]. More specifically, pet as well as service dogs whose owners have epilepsy are exposed to very stressful situations. A case study [108] on pet dogs reported significant adverse health effects that occurred around seizures, leading to the death of animals in three cases from an overwhelming stress reaction. Aggressive behaviors toward humans (owner as well as all people) during the seizure were also reported.

Most reviewed, studies described alerting behaviors without mentioning adverse effects on pets and service dogs. However frequently described behaviors such as anxious barking, whining, close attachment or pacing are classic clinical signs of canine anxiety [115–117]. Such behaviors could, for example, fall into the categories of vocalizing (barks, whines, whimpers), pacing (walks back and forth, does not remain in one place) and remaining by owner (remains near owner, within touching distance).

Considering the exposure of seizure-dogs to such stressful situations, the best training practices have to be applied as well as a very careful selection of dogs for the training program. Positive association with seizure events seems necessary as a basis of training.

### Risk of bias and future directions

The assessments in the reviewed research were predominantly self-reports. This is a common tool to assess individual perceptions related to depression, anxiety, and quality of life following trauma. However, self-reports may lack objectivity, especially concerning the dogs’ performance [46]. Further studies should corroborate self-reported findings with measures that have a lower risk of bias, such as blinded behavioral observation or physiological assessment. For example, in addition to asking a person about seizure frequency and severity, it would also be informative to track patterns of these events via telemetric monitoring devices that can be worn comfortably during the day. Moreover, to assess the capacity of dogs to alert seizures, it will be critical to record alerting behaviors and the subsequent seizure using video-EEG, in order to verify the occurrence of the seizure and to know its time of occurrence.

It is also possible that positive findings are due to a publication bias, whereby negative or non-significant positive findings are filed away rather than published. Another form of potential bias is researcher expectancy bias. This may be particularly salient for studies in which the researchers designed and conducted the study in addition to providing the service dogs. Independence between the research team and the service providers may lend more credibility to study findings.

## Conclusion

This review of scientific papers has revealed that this topic remains poorly investigated. Studies were mainly retrospective anecdotal reports, with surveys that were prone to subjectivity or case studies with a very small sample. Moreover, neither the training status of the dog nor its function were specified in all reviewed studies. It appears that appropriate empirical evidence that dogs can alert or respond to epileptic seizures is still missing; further research is needed to better understand the characteristics of that determine a dog’s suitability, the aspects of reliability and specificity of an alert for different types of seizures, the mechanisms behind the alerts and the nature of the outcomes of using dogs in context of epilepsy.

## Supporting information

S1 Fig PRISMA 2009 checklist. (DOC)

S1 File Appendix: Dog alerting and/or responding to epileptic seizures: A scoping review. Table A: List of the twenty-three studies dealing with seizure-dogs and excluded due to lack of quantitative results.
(DOCX)
