# Supplementary material for: Dog alerting and/or responding to epileptic seizures: A scoping review
Source: PLoS One. 2018 Dec 4;13(12):e0208280. doi: 10.1371/journal.pone.0208280 (PMC6279040; doi:10.1371/journal.pone.0208280)
Supplement: S1 File — Table A: List of the twenty-three studies dealing with seizure-dogs and excluded due to lack of quantitative results. (DOCX) [file pone.0208280.s002.docx]

**Appendix: dog alerting and/or responding to epileptic seizures: a scoping review**

Amélie Catala^1,2^*, Hugo Cousillas^3^, Martine Hausberger^4^, Marine Grandgeorge^1^

^1^ Univ Rennes, Normandie Univ, CNRS, EthoS (Éthologie animale et humaine) - UMR 6552, F-35380 Paimpont, France

^2^ Association Handi’Chiens, 13 Rue de l'Abbé Groult, Paris, France

^3^ Univ Rennes, Normandie Univ, CNRS, EthoS (Éthologie animale et humaine) - UMR 6552, F-35000 Rennes, France

^4^ CNRS, Univ Rennes, Normandie Univ, EthoS (Éthologie animale et humaine) - UMR 6552, F-35380 Paimpont, France

*Corresponding author

E-mail: amelie.catala@univ-rennes1.fr

Among our literature search, 9 articles corresponded to case studies and we summed up 4 of them here to provide additional paths of thinking.

Differing from survey methods, some authors [1] monitored two patients with their “seizure dogs” using continuous computer-assisted EEG in an Epilepsy Care Unit. Results for the first patient were unclear about alerting abilities of the dog. Indeed, he had four seizures while awake and four while sleeping. The SAD was reported as sleeping through seven of these but alerted 2 seconds before the eighth seizure. For other epileptic events, the dog awoke during the seizure and responded to it. For the second patient, the dog alerted 7 minutes prior the only seizure it could see (i.e. being physically present at this moment). Nevertheless, this patient was diagnosed with non-epileptic seizures as there was no EEG change with the seizure. Authors assumed that the warning behavior of the dog could had triggered the non-epileptic seizure.

Other studies in which dogs alert to PNES (though persons thought experiencing epileptic seizures) have been reported. For example, one [2] described the case of a dog said to alert and respond to seizures of its owner by laying on her across its chest prior to and during seizures and fetched help from neighbors after seizures. The dog also responded by the same way to her husband’s seizures although her type of seizure was unclear and no studied here. Video EEG telemetry led to PNES diagnosis for the women patient. Unfortunately, the dog was not present during the monitoring and it is unknown if the recorded events were typical of the patient’s seizures.

Following these works where PNES were implied, a study [3] examined 6 patients from their center with “seizure response service dogs”. They reported that SRD alerted and/or responded to non-epileptic seizures for 4 patients. In addition to demonstrate that an accurate diagnosis is required for persons asking for seizure dogs, this work combined with others highlighted the question of the dog’s seizure discrimination abilities and of the appropriate training. Indeed, though it was not reported here whether they were specially trained dogs, a review [4] stated that it could be linked to the use of actors that would simulate seizures as part of the dogs training program. Then the fact that dog would alert to PNES would not be surprising.

Finally, in a case study [5], it has been reported that a pet dog spontaneously learned to alert prior the onset of seizure of his owner. Authors said that “this has been confirmed by prolonged ambulatory video EEG”. Although details were missing, this is another report (and the most recent) stating that pet dogs would be able to predict seizures.

Table A: List of the twenty-three studies dealing with seizure-dogs and excluded due to lack of quantitative results

| First author | Year | Reference |
| --- | --- | --- |
| Strong | 1999 | [6] |
| Strong | 2000 | [7] |
| Brown | 2001 | [8] |
| Lawson | 2004 | [9] |
| Flegg | 2005 | [10] |
| Ortiz | 2005 | [1] |
| Doherty | 2007 | [2] |
| Krauss | 2007 | [3] |
| Litt | 2007 | [11] |
| Spencer | 2007 | [12] |
| Kirton | 2007 | [13] |
| Mezösi | 2009 | [14] |
| Plowman | 2009 | [15] |
| Di Vito | 2010 | [16] |
| Brown | 2011 | [4] |
| Wells | 2012 | [17] |
| Lyons | 2014 | [5] |
| Freeman | 2015 | [18] |
| Jory | 2016 | [19] |
| Stace | 2016 | [20] |
| Ulate-Campos | 2016 | [21] |
| Zeagler | 2016 | [22] |
| Burton | 2017 | [23] |

**References**

1. Ortiz R, Liporace J. “Seizure-alert dogs”: Observations from an inpatient video/EEG unit. Epilepsy Behav. 2005;6: 620–622. doi:10.1016/j.yebeh.2005.02.012

2. Doherty MJ, Haltiner AM. Wag the dog: skepticism on seizure alert canines. Neurology. 2007;68: 309. doi:10.1212/01.wnl.0000252369.82956.a3

3. Krauss GL, Choi JS, Lesser RP. Pseudoseizure dogs. Neurology. 2007;68: 308–309. doi:10.1212/01.wnl.0000250345.23677.6b

4. Brown SW, Goldstein LH. Can seizure-alert dogs predict seizures? Epilepsy Res. 2011;97: 236–242. doi:10.1016/j.eplepsyres.2011.10.019

5. Lyons P, Bodamer M, Lyons E, Harry L. Seizure alert dog as an effective seizure detection device in refractory symptomatic localisation related epilepsy: a case report. Epilepsy Curr. 2014;14: 1535–7597.

6. Strong V, Brown SW, Walker R. Seizure-alert dogs — fact or fiction? Seizure. 1999;8: 62–65. doi:10.1053/seiz.1998.0250

7. Strong V, Brown SW. Should people with epilepsy have untrained dogs as pets? Seizure. 2000;9: 427–430. doi:10.1053/seiz.2000.0429

8. Brown SW, Strong V. The use of seizure-alert dogs. Seizure. 2001;10: 39–41. doi:10.1053/seiz.2000.0481

9. Lawson SW, Wells D, Strong V. Using support dogs to inform assistive technology: towards an artificial seizure alert system. 2004. Available: https://pure.qub.ac.uk/portal/en/publications/using-support-dogs-to-inform-assistive-technology-towards-an-artificial-seizure-alert-system(056de4dc-1eb5-4290-991a-a47d9e8fa9f6).html

10. Flegg PJ. Seizure-alerting and -response behaviors in dogs living with epileptic children. Neurology. 2005;64: 581; author reply 581.

11. Litt B, Krieger A. Of seizure prediction, statistics, and dogs: a cautionary tail. Neurology. 2007;68: 250–251. doi:10.1212/01.wnl.0000255912.43452.12

12. Spencer DC. Understanding seizure dogs. Neurology. 2007;68: E2-3. doi:10.1212/01.wnl.0000255054.50937.13

13. Kirton A. PSEUDOSEIZURE DOGS. Neurology. 2007;68: 2045. doi:10.1212/01.wnl.0000268590.95483.74

14. Mezősi T, Pallos A, Komondi P, Topál J. Is it reasonable to forbid the use of guide dogs for the blind as predictors of epileptic seizures? A case report. J Vet Behav Clin Appl Res. 2009;4: 85. doi:10.1016/j.jveb.2008.09.038

15. Plowman. “Okay girl, it’s up to you”: a case study of the use of a seizure alert dog to improve the wellbeing of a student with epilepsy [Internet]. 2009 [cited 13 Jan 2017]. Available: https://www.researchgate.net/publication/27484097_Okay_girl_it’s_up_to_you_a_case_study_of_the_use_of_a_seizure_alert_dog_to_improve_the_wellbeing_of_a_student_with_epilepsy

16. Di Vito L, Naldi I, Mostacci B, Licchetta L, Bisulli F, Tinuper P. A seizure response dog: video recording of reacting behaviour during repetitive prolonged seizures. Epileptic Disord Int Epilepsy J Videotape. 2010;12: 142–145. doi:10.1684/epd.2010.0313

17. Wells DL. Dogs as a diagnostic tool for ill health in humans. Altern Ther Health Med. 2012;18: 12–17.

18. Freeman WD, Vatz KA. The Future of Health Care: Going to the Dogs? Front Neurol. 2015;6. doi:10.3389/fneur.2015.00087

19. Jory C, Shankar R, Coker D, McLean B, Hanna J, Newman C. Safe and sound? A systematic literature review of seizure detection methods for personal use. Seizure. 2016;36: 4–15. doi:10.1016/j.seizure.2016.01.013

20. Stace LB. Welcoming max: Increasing pediatric provider knowledge of service dogs. Complement Ther Clin Pract. 2016;24: 57–66. doi:10.1016/j.ctcp.2016.05.005

21. Ulate-Campos A, Coughlin F, Gaínza-Lein M, Fernández IS, Pearl PL, Loddenkemper T. Automated seizure detection systems and their effectiveness for each type of seizure. Seizure. 2016;40: 88–101. doi:10.1016/j.seizure.2016.06.008

22. Zeagler C, Zuerndorfer J, Lau A, Freil L, Gilliland S, Starner T, et al. Canine Computer Interaction: Towards Designing a Touchscreen Interface for Working Dogs. Proceedings of the Third International Conference on Animal-Computer Interaction. New York, NY, USA: ACM; 2016. pp. 2:1–2:5. doi:10.1145/2995257.2995384

23. Burton A. What about a seizure-alert dog? Lancet Neurol. 2017;16: 265–266. doi:10.1016/S1474-4422(17)30050-9
